# Impact of a PCR point of care test for influenza A/B on an acute medical unit in a large UK teaching hospital: results of an observational, pre and post intervention study

**DOI:** 10.1186/s13756-019-0575-6

**Published:** 2019-07-16

**Authors:** Mark I. Garvey, Martyn A. C. Wilkinson, Craig W. Bradley, Martin Biggs, Vinay Reddy-Kolanu, Husam Osman, Sarah Carmalt, Elisabeth Holden

**Affiliations:** 1 University Hospitals Birmingham NHS Foundation Trust, Queen Elizabeth Hospital Birmingham, Edgbaston, Birmingham, B15 2WB UK; 2 Institute of Microbiology and Infection, The University of Birmingham, Edgbaston, Birmingham, UK; 3 Gloucestershire Hospitals NHS Foundation Trust, Gloucestershire Royal Hospital, Gloucester, GL1 3NN UK

**Keywords:** Influenza, Point of care testing, PCR, Acute medical unit, Emergency department

## Abstract

**Background:**

Influenza viruses is a leading cause of acute respiratory infection, placing a significant burden on healthcare. To reduce hospital transmission, patients clinically suspected of having influenza are isolated and offered empirical antiviral treatment. Here we report the use of a point of care test (POCT) for influenza viruses in an acute medical unit (AMU) at Queen Elizabeth Hospital Birmingham for patients presenting with influenza-like illness.

**Methods:**

A PCR POCT was installed on AMU in Dec 17 – Mar 18 (period 2) and used to test any patient with influenza-like illness. We conducted an evaluation against influenza virus’s data collected between Dec 16–Mar 17 (period 1) where no POCT was used. Four outcomes were measured: length of stay, oseltamivir utilisation, time to isolation and in-hospital cases of influenza viruses.

**Results:**

There were 51 confirmed influenza virus cases in period 1 vs 666 in period 2. During period 2, the length of stay of patients presenting with influenza-like illness (2.4 vs 7.9 days) and time to isolation from receipt of a positive result (0.09 vs 1.26 days) was significantly shorter. The time to initial receipt of antivirals for patients with influenza virus was significantly quicker in period 2 (0.59 vs 1.1 days) and the total number of influenza virus cases identified after 72 h of admission was significantly lower (9% vs 51%).

**Discussion:**

Following introduction of the POCT, there was an increase in appropriately targeted oseltamivir prescribing, shorter time to isolation, proportionally less post-72-h influenza virus cases and a reduction in length of stay of patients presenting with influenza-like illness.

**Conclusions:**

Routine use of POCTs for viruses should be introduced into diagnostic pathways for acute respiratory illness, especially at the front door of hospitals.

## Background

Influenza viruses is a leading cause of acute respiratory infection. It is responsible for a large burden of disease, including serious complications in patients with risk factors such as pregnancy, young children, elderly, or those with underlying medical conditions [1, 2]. Studies have shown respiratory viruses are detectable in 40–50% of hospitalised adults with acute respiratory illness [3, 4]. The case fatality rate of influenza in hospitalised patients is quoted at 3–8% [3, 5]. Influenza viruses are highly transmissible within both community and healthcare settings, and places a significant burden on healthcare [1–3]. Nosocomial influenza virus outbreaks lead to increased bed occupancy and closed wards during winter months, with significant financial implications [6]. Prevention of influenza virus transmission within healthcare facilities requires a multipronged approach, including general precautions such as correct hand hygiene, respiratory etiquette, patient specific contact/droplet precautions, vaccination of patients and staff, antiviral treatment or chemoprophylaxis and surveillance of cases [7, 8]. Rapid detection and implementation of chemoprophylaxis within hospitals has been identified as one of the most important interventions to contain an influenza virus outbreak [9].

Influenza virus testing in the UK is based on clinical suspicion and the use of laboratory-based PCR tests [10, 11]. In the UK, laboratory-based PCR turnaround times range at 24–48 h [10, 12]. One of the mainstays of controlling influenza in healthcare is around timely diagnosis [10]. Point of care tests (POCT) can be non-molecular or molecular-based [13]. POCT for influenza viruses have been previously antigen-based, however lack sensitivity [13]. More recently, rapid molecular tests are available and broadly equivalent to laboratory-based PCR [13]. A major benefit of POCT is the rapidity of results, which is important to guide clinical management [13]. They can also guide infection control measures to optimise patient allocation and bed utilisation within the hospital, reduce nosocomial transmission of respiratory viruses and provide an earlier diagnosis of viral respiratory tract infections [13]. An influenza virus POCT, specifically a Cepheid GeneXpert, was used in the acute medical unit (AMU) at Queen Elizabeth Hospital Birmingham (QEHB), part of University Hospitals Birmingham (UHB) NHS Foundation Trust, during the 2017/18 influenza season. After the end of the influenza season, a service evaluation was conducted to assess the impact of the POCT on patient care and infection control. The results were compared to the previous influenza season (2016/17), when no POCT was used. Outcomes included rates of healthcare-associated influenza virus, utilisation of oseltamivir, length of stay and achievement of respiratory isolation.

## Materials and methods

### Setting

QEHB is a major UK teaching hospital, providing clinical services to nearly 1 million patients every year. The AMU at QEHB consists of an Emergency Department and a Clinical Decision Unit with a combined total of 88 beds.

### Time period

The evaluation was conducted at QEHB in patients presenting with influenza-like illness during two time periods (period 1 Dec 16–Mar 17 vs period 2 Dec 17-Mar 18). A PCR POCT (Cepheid, USA; GeneXpert) was installed on AMU in period 2 and used to test any patients with influenza-like illness. In period 1 a laboratory-based PCR (Cepheid, USA; GeneXpert) was used to test for influenza A, influenza B, respiratory syncytial virus (RSV). The GeneXpert was used as previously described and tests for Influenza A/B and RSV [14]. The PCR POCT was housed within a store room within AMU, easily accessible by all staff on AMU. The staff that used the POCT were the ward nurses, trained by the in house POCT team. The POCT test was validated and quality control checked by the POCT team comparing to the in house laboratory based PCR test.

### Outcomes

Four outcomes were used to assess the impact of the POCT: length of stay, drug utilisation (oseltamivir prescription), time to isolation after receipt of a positive result and healthcare associated influenza virus cases.

### Data collection

The Infection Prevention and Control team kept a database of all positive respiratory virus results between Sept 2016 to June 2018. The database was cross referenced against PCR data extracted from the Laboratory Information Management system and the POCT used on AMU to ensure all positive respiratory virus results were picked up. The database was populated from QEHBs Patient Information and Communication System with: patient details, date of isolation, date of the influenza virus result, date of admission and start date of the oseltamivir prescription. Community-acquired influenza infection was defined as influenza virus detection by PCR < 72 h after admission, for healthcare-associated influenza infection this was defined as influenza virus detection by PCR ≥ 72 h after admission [3]. This standard definition is based upon the usual in-vivo incubation period for influenza being 1–3 days and a practical solution to the fact that information pertaining to symptom onset was not readily available [3, 15, 16].

### Statistical analysis

All admission and discharge dates were retrieved from the electronic admission records system by the hospital informatics service. Non–parametric statistical tests were used to analyse the effect of the POCT on length of stay. Using length of stay as the response variable, patients admitted to QEHB during period 1 (2016/17) and period 2 (2017/18) were divided into different groups based on the clinical coding of patients on admission (Table 1). The clinical coding of patients admitted to QEHB who had confirmed and/or suspected influenza fell into two categories; lower respiratory tract infection and influenza, and influenza alone (Table 1). To note all patients presenting with acute respiratory tract infection would have been tested for Influenza infection. A Kruskal-Wallis test was used to see whether there was evidence of disparity in the median length of stay for the different groups. A pairwise comparisons amongst the groups, looking for evidence that the medians were disparate was undertaken. The tests used here were Mann-Whitney U tests, with Holm’s correction for multiple comparisons.

**Table 1:** Length of stay data in period 1 (2016/17) and period 2 (2017/18) from patients admitted to QEHB with influenza-like illness. All patients with confirmed influenza in periods 1 and 2 fell within these admission coding descriptions

Group	Period	Year	Number of patients	Median LoS (days)	Median LoS (hours)
A - LRTI and Influenza	1	2016/17	10	9.01	216.18
B - Influenza	1	2016/17	49	8.61	206.75
C - LRTI and Influenza	2	2017/18	24	5.16	123.83
D - Influenza	2	2017/18	642	3.75	89.94

Key: LRTI, lower respiratory tract infections; LoS, length of stay

Note: All admission and discharge dates were retrieved from the electronic admission records system by the hospital informatics service. All patients with influenza during period 1 and 2 fell into the admission coding LRTI and Influenza or Influenza alone

A chi-squared test was used to check for equality of proportions between the numbers of healthcare-associated cases of influenza virus in period 1 and 2. A non-parametric test was used measure the difference in means between the time required to isolate patients and the time to prescribe oseltamivir in period 1 and 2 as the data was non-normal. As such a Mann-Whitney U test with continuity correction was used.

### Ethics

No IRB approval was needed (exemption).

## Results

### QEHB influenza seasons

In period 2 there were 256 laboratory confirmed cases of influenza A and 408 laboratory cases of influenza B, of which two patients were dually infected with both viruses. This was significantly more than previous years, where QEHB saw 51 cases in 2016/17 (period 1), 71 cases in 2015/16 and 155 cases in 2014/15.

### Length of stay

**Fig. 1:**
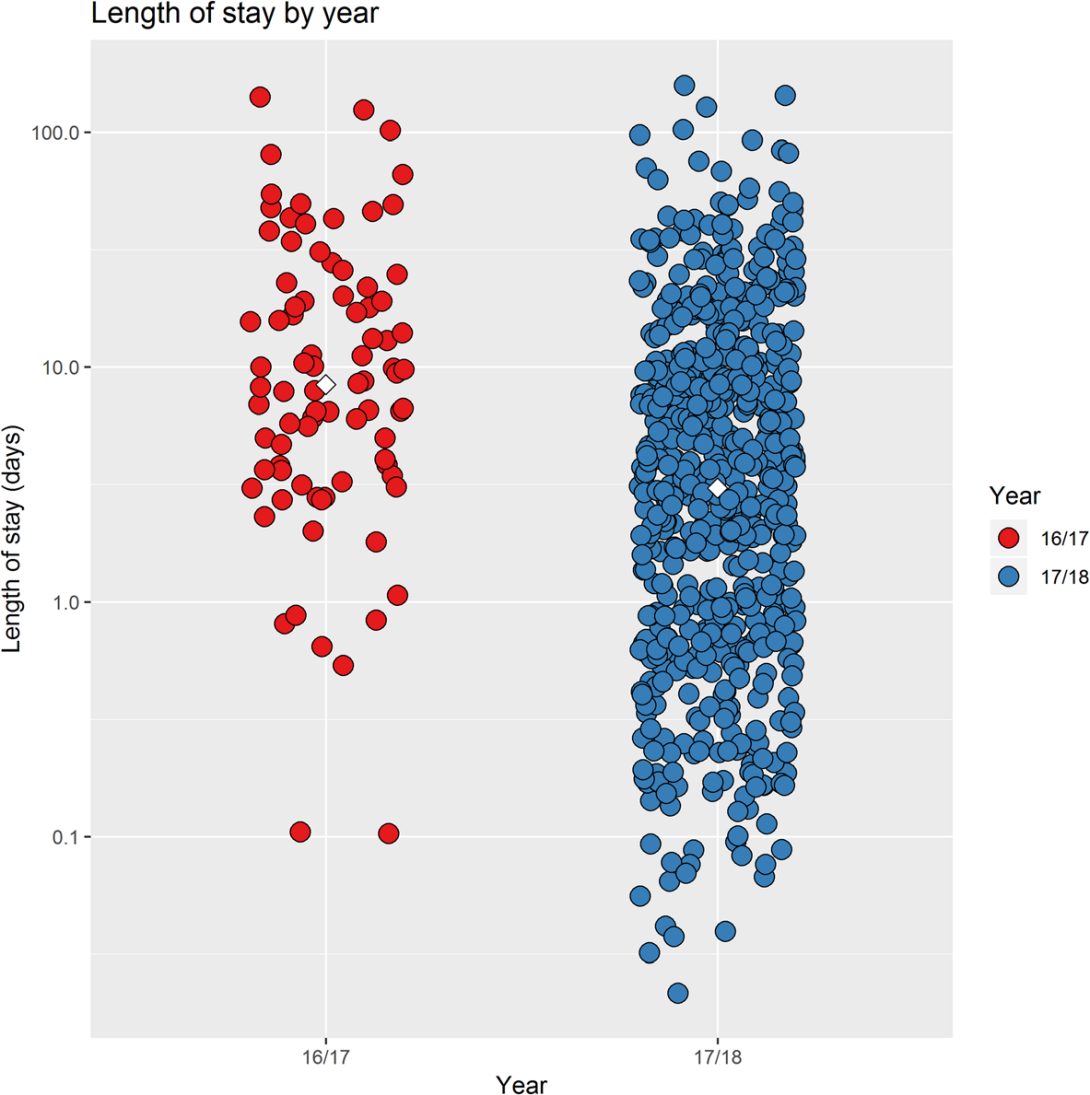
Length of stay in days of all patients with confirmed influenza during period 1 and period 2. Note: The white diamonds represent the mean days of length of stay in period 1 and 2. Red circles represents all patients in period 1. Blue circles represent all patients in period 2

Patients with confirmed/suspected influenza in periods 1 and 2 were divided into different groups based on the clinical coding reason for admission. The categories were lower respiratory tract infection and influenza, and influenza alone (Table 1). A Kruskal-Wallis test was used, to see whether there was evidence of disparity in the median length of stay data for the different groups. The *p* < 0.001 (*p* = 2.20 × 10^− 16^) provided strong evidence that the medians differ. A pairwise comparison was performed amongst the groups, looking for evidence that the medians were disparate. A Mann-Whitney U test, with Holm’s correction for multiple comparisons was used on the groups (Table 2). Patients coded with influenza alone in period 1 (2016/17) had a median length of stay was 206.75 h as compared to period 2, (2017/18) where the median length of stay of 89.94 h (Fig. 1). This difference was highly significant *p* < 0.001 (*p* = 1.51 × 10^− 5^).

**Table 2:** Statistical comparison of the length of stay data (days) in period 1 (2016/17) and period 2 (2017/18) of patients admitted to QEHB with influenza-like illness, using a Mann Whitney U test, with Holm’s correction for multiple comparisons

Comparison	Median 1	Median 2	*p*-value
D vs C	3.75	5.16	0.183
D vs B	3.75	8.61	1.59 × 10^−6^
D vs A	3.75	9.01	0.166
C vs B	5.16	8.61	0.535
C vs A	5.16	9.01	0.720
B vs A	8.61	9.01	0.812

Key: A, lower respiratory tract infection and influenza in period 1; B, influenza in period 1; C, lower respiratory tract infection and influenza in period 2; D, influenza in period 2

Note: A Kruskal-Wallis test was used on the data, to see whether there was evidence of disparity in the median length of stay for the different groups. Next, pairwise comparisons amongst the groups, looking for evidence that the medians were disparate was performed. The tests used here were Mann-Whitney U tests, with Holm’s correction for multiple comparisons. The table displays the results for the comparisons

### Time to isolation

**Fig. 2:**
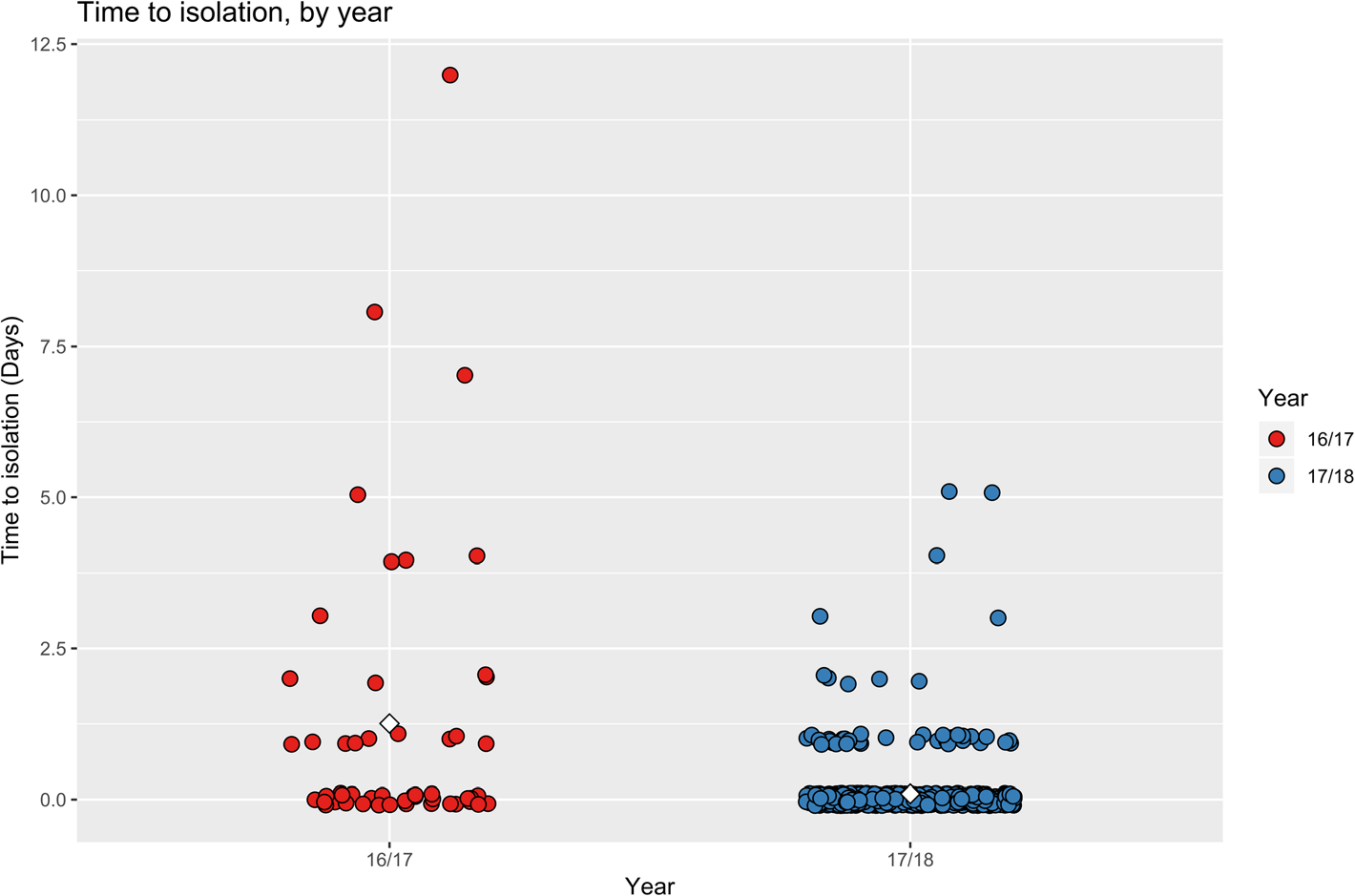
Time to isolation in days of all patients with confirmed influenza during period 1 and period 2. Note: The white diamonds represent the mean days till isolation in period 1 and 2. Red circles represents all patients in period 1. Blue circles represent all patients in period 2

Of the 666 patients with either Influenza A or B in period 2, the mean time to isolation after receipt of a positive result was 0.09 of a day (median 0 days) (Fig. 2). Compared to period 1 this was shorter, where the mean time to isolation was 1.25 days (median 1 day) (Fig. 2). A Mann-Whitney U test was used to measure the difference in means between period 1 and 2. The *p* < 0.001 (*p* = 2.2 × 10^− 16^) provided strong evidence that the means differ.

### Administration of antivirals

**Fig. 3:**
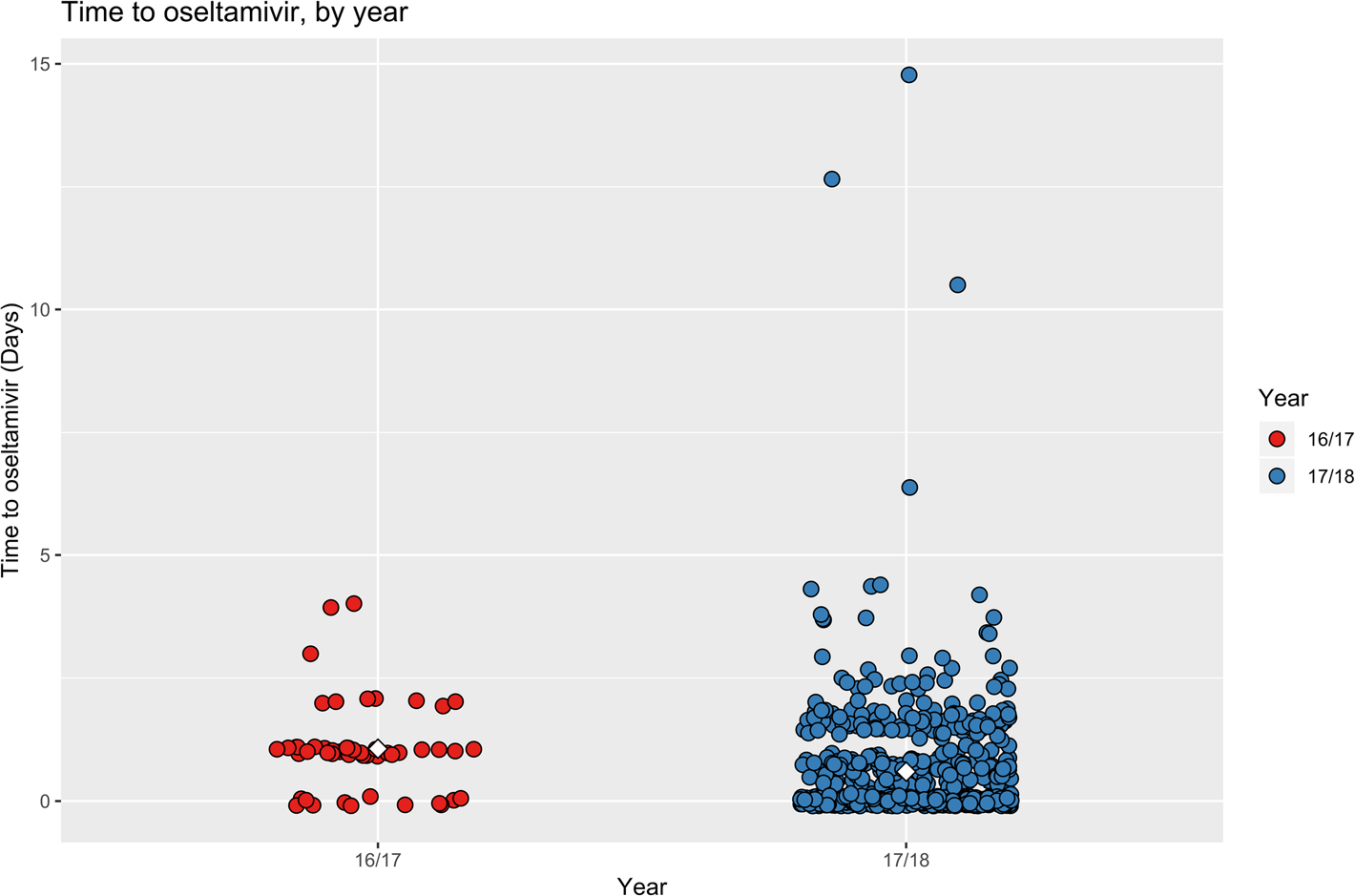
Time to first dose of oseltamivir (days) in all patients with confirmed influenza during period 1 and period 2. Note: The white diamonds represent the mean days till first dose of oseltamivir in period 1 and 2. Red circles represents all patients in period 1. Blue circles represent all patients in period 2

The time to initial receipt of antivirals for patients with influenza virus was quicker in period 1, with a median of 0.6 days; compared to period 1, with a median of 1.06 days (Fig. 3). A Mann Whitney U test with continuity correction was used measure the difference in means between period 1 and 2 (Fig. 3). A p < 0.001 (*p* = 1.058 × 10^− 7^) provided strong evidence that the means differ.

### Rates of healthcare associated influenza virus

In period 1 25 of the 51 cases (49%) were identified after 72 h of admission. Fewer healthcare associated influenza virus were identified in period 2 with 64 of the 666 cases (9%) identified after 72 h of admission. A chi squared test was used to check for equality of proportions between period 1 and 2. A p < 0.001 (*p* = 1.184 × 10^− 15^) provided very strong evidence that the proportions differ. The 95% confidence interval for the difference in the two proportions was (0.2445, 0.5437).

## Discussion

Influenza virus is highly transmissible in a healthcare setting. As such it is recommended that patients clinically suspected of having influenza are isolated and offered empirical antiviral treatment [1, 2]. Rapid influenza virus detection and implementation of chemoprophylaxis within hospitals has been identified as one of the most important interventions to contain an outbreak [1]. Recently, Young et al.*,* (2018) discussed the impact of a POCT for influenza virus in an emergency department [3]. The POCT was associated with reduced nosocomial transmission of influenza virus and improved patient flow [3]. A recent randomised clinical trial showed similar effects and suggested use of a POCT was associated with a reduced length of stay as well as improved influenza virus detection and antiviral use [10]. Here, we report our experience of using a POCT in an AMU looking primarily at four outcomes: length of stay, oseltamivir utilisation, time to isolation and in-hospital cases of influenza virus.

At QEHB, healthcare associated influenza virus cases of 49% (period 1) were reported in 2016/17 compared to 9% (period 2) in 2017/18. There was strong statistical evidence that the mean number of healthcare associated influenza virus cases differed between period 2 where the POCT was used, compared to period 1 where it was not used. Young et al.*,* (2018) recently demonstrated a similar result where the rate of healthcare-associated infection per day was lower after the implementation of a POCT in an emergency department [3]. The number of healthcare associated influenza virus cases in period 2 when the POCT was introduced in our setting could be in part due to the POCT. Young et al.*,* (2018) demonstrates that a POCT enables influenza virus cases to be identified earlier, thereby allowing for appropriate infection control precautions such as isolation and antiviral treatment [3]. In addition, patients presenting with influenza could be discharged home more quickly, thereby prevented delayed diagnosis within the healthcare setting and the opportunity for transmission [3, 10]. Similarly in a recent RCT by Brendish et al.*,* (2017) comparing the use of a POCT vs an in-house laboratory method, a reduction in length of stay was observed in patients testing positive for respiratory viruses [10]. Brendish et al.*,* (2018) also demonstrated rapid turnaround times using a POCT are associated with higher rates of early discharge and early discontinuation of antibiotics compared to longer turnaround times in adults with acute respiratory illness [17]. Other reasons for the results observed in the current study could include the acuity of the strains of influenza virus, which could potentially have been less virulent. Out of the 666 cases of influenza viruses reported in period 2, 408 cases were influenza B, as compared to period 1, where there were three cases of influenza B. Further work is warranted to observe whether a POCT does in fact reduce transmission rates via more timely identification.

The reduction in healthcare-associated influenza virus cases seen in the current study may result from improvements in respiratory isolation. The ability for respiratory isolation in the first five days of admission when patients are most infectious has been previously shown to reduce rates of transmission of healthcare-associated influenza viruses. Time to isolation in period 2 was significantly quicker compared to period 1 in the current study; the POCT could in part explain these results. Brendish et al.*,* (2017) demonstrated side room isolation for confirmed respiratory virus infection was more common when a POCT was used [10]. Similarly to our study, they demonstrated better use of side rooms when the POCT was used; with reduced time from admission to isolation in confirmed influenza virus cases [10]. The authors concluded that rapid and appropriate assignment of side rooms for patients with respiratory virus infection is hugely important to reduce the risk of nosocomial transmission to other vulnerable hospitalised patients [10]. Similar findings would be true at QEHB where patients with transmissible respiratory viral infections are isolated (data not shown). When the infection status is not known, or even considered, patients are not often isolated. Young et al.*,* (2018) demonstrated that when a POCT was not used to identify patients with influenza they were not isolated as often as than those patients where a POCT was used to diagnose influenza (21.5% vs 74.8%). Thereby increasing the risk of influenza virus exposure to susceptible patients [3].

In addition to reductions in healthcare associated influenza virus cases and time to isolation, our study shows that POCT might be associated with a reduction in hospital length of stay. The median length of stay of patients with influenza virus in period 2 was statistically lower compared to period 1. It is possible that the prevalent strain of influenza virus provoked less severe disease in period 2 than period 1, which could in part explain the results seen. Brendish et al.*,* (2017) demonstrated a similar finding in patients with exacerbation of airways disease [10]. The authors concluded that reduced hospital length of stay was due to earlier discharge in patients testing positive for respiratory viruses via a POCT [10]. The reduction in length of stay reported by Brendish et al.*,* (2017) was in the order of 1 day, which equated to around 200,000 bed days saved, with a cost saving of £80 million per year [10, 18]. In the current study, the reduction in length of stay was in the magnitude of 3 days, with a higher proportion of bed days saved and thus the potential for even greater cost savings. Further work is warranted to explore the cost savings.

In agreement with prior studies, we found that prescription of oseltamivir increased post-introduction of an influenza virus POCT (data not shown) [19–21]. Oseltamivir treatment is recommended by UK Public Health England for all patients hospitalised with influenza [22]. Our study showed a statistically quicker administration of oseltamivir in period 2, when a POCT was used, as compared to period 1. Increased oseltamivir prescription may have contributed towards a reduction in healthcare-associated influenza viruses seen at QEHB by reducing ongoing transmission. Brendish et al.*,* (2017) showed that a POCT for respiratory viruses leads to an increased proportion of influenza-positive patients correctly receiving treatment with neuraminidase inhibitors and suggested a reduced time to administration of the first dose [10]. There is much debate on the effectiveness of oseltamivir, however the literature details the most effective use is within the first 48 h of symptoms [23]. The POCT in our setting certainly helped with quicker administration of oseltamivir. Further work is warranted to look at the effect of neuraminidase inhibitors on resolution of symptoms and the effectiveness within the first 48 h of treatment.

Finally unlike Brendish et al.*,* (2017) this study used an influenza virus/RSV POCT rather than syndromic multiple for respiratory viruses [10]. The added value for syndromic multiplex molecular POCT above molecular Influenza virus testing is currently unknown but there are potential clinical benefits to the detection of other non-influenza viruses at the POCT including infection control and early cessation of stopping unnecessary antibiotics, although these would need to be considered against the extra expense of the syndromic panels.

Limitations of the study include the differences in Influenza season in periods 1 and 2. There was a large difference in the amount of Influenza virus seen and a difference in Influenza strains seen. This could have affected the results observed and warrants further analysis in future Influenza seasons. Another factor to explain the results seen is that the acuity of the strains of influenza could potentially have been less virulent in the current study. However, despite the limitations the quality and efficiency of management of influenza-like illness was improved in season 2. To note there were no other interventions during these two periods 1 and 2.

## Conclusion

In conclusion, following the introduction of the POCT at QEHB, there was an increase in appropriately targeted oseltamivir prescribing, shorter time to isolation, proportionally less 72-h influenza virus cases and a reduction in length of stay of patients presenting with influenza-like illness. This study demonstrates that POCTs have the potential to improve the quality and efficiency of the management of influenza-like illness. Although difficult to quantify, there may be an additional benefit of admission avoidance. As per Brendish et al.*,* (2017) influenza virus POCT seems to be associated with health economic benefit [10]. Routine use of POCTs for viruses should be introduced into diagnostic pathways for acute respiratory illness, especially at the front door of hospitals.

## Data Availability

Not applicable.

## Abbreviations

AMU: Acute Medical Unit
PCR: Polymerase Chain Reaction
POCT: Point of care test, a laboratory test used at the pit of care
QEHB: Queen Elizabeth Hospitals Birmingham
UHB: University Hospitals Birmingham NHS Foundation Trust

## References

[1] Reed Carrie, Chaves Sandra S., Daily Kirley Pam, Emerson Ruth, Aragon Deborah, Hancock Emily B., Butler Lisa, Baumbach Joan, Hollick Gary, Bennett Nancy M., Laidler Matthew R., Thomas Ann, Meltzer Martin I., Finelli Lyn (2015). Estimating Influenza Disease Burden from Population-Based Surveillance Data in the United States. PLOS ONE.

[2] Nair H, Nokes DJ, Gessner BD, Dherani M, Madhi SA, Singleton RJ (2010). Global burden of acute lower respiratory infections due to respiratory syncytial virus in young children: a systematic review and meta-analysis. Lancet.

[3] Youngs J., Iqbal Y., Glass S., Riley P., Pope C., Planche T., Carrington D. (2019). Implementation of the cobas Liat influenza point-of-care test into an emergency department during a high-incidence season: a retrospective evaluation following real-world implementation. Journal of Hospital Infection.

[4] Clark TW, Medina MJ, Batham S, Curran MD, Parmar S, Nicholson KG (2014). Adults hospitalised with acute respiratory illness rarely have detectable bacteria in the absence of COPD or pneumonia; viral infection predominates in a large prospective UK sample. J Inf Secur.

[5] Lee N, Ison MG (2012). Diagnosis, management and outcomes of adults hospitalized with influenza. Antivir Ther.

[6] Bouscambert M, Valette M, Lina B (2015). Rapid bedside tests for diagnosis, management, and prevention of nosocomial influenza. J Hosp Infect.

[7] Public Health England (2016). Infection control precautions to minimise transmission of respiratory tract infections in healthcare settings.

[8] Centers for Disease Control and Prevention. Prevention strategies for seasonal influenza in healthcare settings. Atlanta, GA: CDC; 2018. Available at: https://www.cdc.gov/flu/professionals/infectioncontrol/healthcaresettings.htm (last accessed Feb 2010).

[9] Lowe CF, Leung V, Karakas L, Merrick L, Lawson T, Romney MG (2019). Targeted Management of Influenza a/B outbreaks incorporating the cobas® influenza a/B & RSV into the virology laboratory. J Hosp Infect.

[10] Brendish NJ, Malachira AK, Armstrong L, Houghton R, Aitken S, Nyimbili E (2017). Routine molecular point-of-care testing for respiratory viruses in adults presenting to hospital with acute respiratory illness (ResPOC): a pragmatic, open-label, randomised controlled trial. Lancet Respir Med.

[11] Elf S, Auvinen P, Jahn L, Likonen K, Sjoblom S, Saavalainen P (2018). Development and evaluation of a rapid nucleic acid amplification method to detect influenza A and B viruses in human respiratory specimens. Diag Micro & Infect Dis.

[12] Brendish NJ, Schiff HF, Clark TW (2015). Point-of-care testing for respiratory viruses in adults: the current landscape and future potential. J Inf Secur.

[13] Public Health England, Point of care tests for Influenza and other respiratory viruses, November 2018 available at: https://assets.publishing.service.gov.uk/government/uploads/system/uploads/attachment_data/file/762344/point_of_care_tests_for_influenza_and_other_respiratory_viruses.pdf (last accessed 13 February 2019).

[14] Novak-Weekley SM, Marlowe EM, Poulter M, Dwyer D, Speers D, Rawlinson W, Baleriola C, Robinson CC. Evaluation of the cepheid Xpert Flu assay for rapid identification and differentiation of Influenza A 2009 H1N1, and Influenza B viruses. J Clin Microbiol. 50(5):1704–10. 10.1128/JCM.06520-11 PMC3347140 22378908

[15] Salgado CD, Farr BM, Hall KK, Hayden FG (2002). Influenza in the acute hospital setting. Lancet Infect Dis.

[16] Public Health England, Department of Health. Influenza. Green Book 2017:1–26.

[17] Brendish NJ, Malachira AK, Beard KR, Ewings S, Clark TW (2018). Impact of turnaround time on outcome with point-of-care testing for respiratory viruses: a post-hoc analysis from a randomised controlled trial. Eur Respir J.

[18] Department of Health. Reference costs guidance 2015–16. February, 2016. https://assets.publishing.service.gov.uk/government/uploads/system/uploads/attachment_data/file/497127/Reference_costs_guidance_2015-16.pdf (accessed 13 Feb 2019).

[19] Egilmezer E, Walker GJ, Bakthavathsalan P, Peterson JR, Gooding JJ, Rawlinson W, et al. Systematic review of the impact of point of care testing for influenza on the outcomes of patients with acute respiratory tract infection. Rev Med Virol. 2018; e1995. 10.1002/rmv.1995 PMC7169080 30101552

[20] Chu HY, Englund JA, Huang D (2015). Impact of rapid influenza PCR testing on hospitalization and antiviral use: a retrospective cohort study. J Med Virol.

[21] Vecino-Ortiz AI, Goldenberg S, Douthwaite ST, Cheng CY, Glover RE, Mak C (2018). Impact of a multiplex PCR point of care test for influenza a/B and respiratory syncytial virus on acute pediatric hospital ward. Diagn Microbiol Infect Dis.

[22] Public Health England (2016). PHE guidance on use of antiviral agents for the treatment and prophylaxis of Influenza.

[23] Muthuri SG, Venkatesan S, Myles PR (2014). Effectiveness of neuraminidase inhibitors in reducing mortality in patients admitted to hospital with influenza A H1N1pdm09 virus infection:a meta-analysis of individual participant data. Lancet Respir Med.

